# All the right moves: why *in utero* transfer is both important for the baby and difficult to achieve and new strategies for change

**DOI:** 10.12688/f1000research.25923.1

**Published:** 2020-08-13

**Authors:** Helena Watson, James McLaren, Naomi Carlisle, Nandiran Ratnavel, Tim Watts, Ahmed Zaima, Rachel M. Tribe, Andrew H. Shennan

**Affiliations:** 1 Guy’s and St Thomas NHS Foundation Trust, London, UK; 2 Department of Women and Children’s Health, School of Life Course Sciences, King’s College London, London, UK; 3 Gosford Hospital, Gosford, New South Wales, Australia; 4 Royal London Hospital, London, UK; 5 Kingston Hospital, Kingston upon Thames, UK

**Keywords:** in-utero transfer, preterm birth

## Abstract

The best way to ensure that preterm infants benefit from relevant neonatal expertise as soon as they are born is to transfer the mother and baby to an appropriately specialised neonatal facility before birth (“ *in utero*”). This review explores the evidence surrounding the importance of being born in the right unit, the advantages of *in utero* transfers compared to *ex utero* transfers, and how to accurately assess which women are at most risk of delivering early and the challenges of *in utero* transfers.

Accurate identification of the women most at risk of preterm birth is key to prioritising who to transfer antenatally, but the administrative burden and pathway variation of *in utero* transfer in the UK are likely to compromise optimal clinical care. Women reported the impact that *in utero* transfers have on them, including the emotional and financial burdens of being transferred and the anxiety surrounding domestic and logistical concerns related to being away from home. The final section of the review explores new approaches to reforming the *in utero* transfer process, including learning from outside the UK and changing policy and guidelines. Examples of collaborative regional guidance include the recent Pan-London guidance on *in utero* transfers. Reforming the transfer process can also be aided through technology, such as utilising the CotFinder app.

*In utero* transfer is an unavoidable aspect of maternity and neonatal care, and the burden will increase if preterm birth rates continue to rise in association with increased rates of multiple pregnancy, advancing maternal age, assisted reproductive technologies, and obstetric interventions. As funding and capacity pressures on health services increase because of the COVID-19 pandemic, better prioritisation and sustained multi-disciplinary commitment are essential to maximise better outcomes for babies born too soon.

## Introduction

As neonatology has advanced and subspecialised, there has been an impetus to ensure babies have the opportunity to benefit from the relevant expertise as soon as they are born. This is particularly relevant in the initial care of the extremely preterm baby (born <27 weeks’ gestation) ^1^, where evidence shows that care in specialist neonatal intensive care units (NICUs) significantly improves survival ^2– 6^. In the largest relevant UK study to date, perinatal mortality rates were 19% higher for the extremely preterm infants born outside NICUs ^2^. *In utero* transfer of the mother to an appropriately specialised neonatal facility prior to birth is the best way to address this access to care. Given current funding pressures and the expense of neonatal services, capacity issues also contribute to the need to transfer mothers and infants between facilities. In the UK, the prevalence of *in utero* transfer is unknown. It was estimated to be 1.4% by a Finnish birth register study analysing antenatal care bed days between 22 and 32 weeks’ gestation. Even at this relatively low prevalence, given the lifelong health, social, and economic consequences of neonatal morbidity and an obligation to provide equity of expertise to this vulnerable population, the authors believe this is a major public health issue.

Pressures around cot capacity are compounded by rising birth and preterm birth rates, which exceed investment. Since around 90% of *in utero* transfers are for threatened or actual preterm labour, accurate identification of the women most at risk of preterm birth is key to prioritising who to transfer antenatally. This review will explore the evidence surrounding why *in utero* transfers are important, the challenges faced by clinicians, and the impact on women.

## Why do we need *in utero* transfer?

### The importance of being born in the right facility

There is good evidence that the benefits of being born in a centre with a NICU (also called a ‘level 3 neonatal unit’ or ‘tertiary neonatal unit’) are most significant at early gestational ages ^2– 5^. Every country has specific rules regarding how neonatal care is organised which affect transfer guidelines, particularly around infants at 27–32 weeks’ gestation. In the UK, level 3 neonatal units provide care for all babies at gestations after 22 ^+6^ weeks and level 2 units provide care from 26 ^+6^ weeks gestational age (singletons) and 27 ^+6^ weeks (multiple births) providing the anticipated birth weight is above 800 g. Level 1 units usually provide care after 31 ^+6^ weeks gestational age provided the anticipated birth weight is above 1,000 g ( Table 1 and Table 2).

**Table 1.** Description of categories of neonatal care.

Intensive care	Specialised care for the smallest and most seriously ill babies who require constant care and often mechanical ventilation to keep them alive. Babies with severe respiratory disease and those who also require surgery will need this level of care too.
High-dependency care	Care provided to babies who need continuous monitoring. For babies needing non-invasive breathing support, including receiving continuous positive airway pressure. Babies receiving parenteral nutrition (intravenous feeding) also need this level of care.
Special care	The least-intensive level of neonatal care and the most common. For babies who need continuous monitoring of their breathing or heart rate, additional oxygen, tube feeding, phototherapy (to treat neonatal jaundice), and convalescence from other care.
Transitional care	Babies who have special care needs (e.g. low birth weight, neonatal abstinence syndrome) but are able to be managed alongside the mother as the main carer, supported by neonatal and midwifery teams. In some services, transitional care occurs in the postnatal ward and, in others, in a discreet area or transitional care unit with staffing from both neonatal and midwifery teams.

This table was adapted from NHS England service specifications ^7^ under the terms of the Open Government Licence v3.0.

**Table 2.** Definitions of levels of neonatal unit, stratified by the level of neonatal care they provide.

**Level 1** **Special Care Unit** **(SCU)**	Commissioned and staffed to provide care for babies of births after 31 ^+6^ weeks’ gestation provided the anticipated birth weight is above 1,000 g. Some Operational Delivery Networks have approved care pathway where babies born between 30 ^+0^ and 31 ^+6^ weeks’ gestation receive initial care in the SCU provided the anticipated birth weight is above 1,000 g and intensive care is not required. Some SCUs will provide care only for babies >33 ^+6^ weeks’ gestation.
**Level 2** **Local Neonatal** **Unit (LNU)**	Commissioned and staffed to provide care for babies of singleton births after 26 ^+6^ weeks’ gestation and multiple births after 27 ^+6^ weeks’ gestation providing the anticipated birth weight is above 800 g.
**Level 3** **Neonatal** **Intensive Care** **Unit (NICU)**	Commissioned and staffed to provide care for all babies from birth, in line with national guidelines and professional standards, at all gestations after 22 ^+6^ weeks. All level 3 NICU services will also provide lower-level neonatal support across their maternity catchment area.

This table was adapted from NHS England service specifications ^7^ under the terms of the Open Government Licence v3.0.

The development of neonatal networks in the UK from 2004 was aimed at ensuring babies who required intensive care after birth were cared for in properly accredited, staffed, and equipped NICUs, and it was recognised even then that it was particularly important in those born extremely preterm (NHS England, 2015). The networks also facilitated repatriation once the neonate was well enough to receive the appropriate level of care locally. Despite this, the most recent UK national cohort (EpiCure 2) revealed that only 56.4% (1,387/2,640) of births between 22 and 26 weeks’ gestation occurred in hospitals with a level 3 NICU ^2^. Perinatal mortality rates at this gestation were 72% in a level 1 unit compared with 53% in a NICU facility ( *P* <0.0001). Excluding the antenatal deaths, early neonatal deaths were also significantly higher for those delivered at a level 1 versus level 3 facility (33% versus 23%, *P* <0.0001) ^2^. Another recent UK study reported an increased mortality (odds of death 1.34, confidence interval [CI] 1.02–1.77; number needed to treat [NNT] 20) for those born in a level 2 neonatal unit compared with a level 3 unit ^8, 9^.

Similar findings have been reported outside the UK. A population cohort study from Victoria, Australia, analysed 541 livebirths (excluding congenital malformations) between 22 ^+0^ and 27 ^+6^ weeks’ gestation ^10^. They compared those born in a level 3 neonatal unit with those born outside of level 3 units directly. Even excluding infants born at 22 weeks’ gestation from the analysis, overall mortality for outborn infants was higher than that of inborn counterparts (adjusted odds ratio [paOR] 2.70, 95% CI 1.49–4.92, *P* <0.001) ^10^. In a Swedish population study of extremely low birth weight infants, mortality was also found to be directly proportional to the level of care received ^11^. An international meta-analysis of the effect of perinatal regionalisation reported a 62% increase in the odds of neonatal mortality for very low birth weight (<1,500 g) infants born in a non-level 3 facility (37 articles, 104,944 infants) and a 55% increase in the odds of mortality for very preterm infants (<32 weeks) ^5^.

Despite the body of evidence in favour of birth in a unit with appropriate neonatal expertise, not all mothers are accessing this care. In the EPICE population cohort, 12% of infants were not born in the correct facility, and these infants were also less likely to receive other evidence-based practices such as antenatal corticosteroids, prevention of hypothermia, early surfactant, or continuous positive airway pressure use ^4^.

### Ex utero versus in utero transfer

There is broad consensus favouring birth in units with appropriate NICU expertise for very premature infants, but when mothers present elsewhere does transfer before or after birth make such a difference? Comparison of *ex utero* transfer (usually immediate but may be up to 48 hours postnatally) and *in utero* transfer is usually achieved by retrospective comparison of outcomes of NICU infants born in-house compared with those transferred in. Most studies confirm reductions in morbidity and mortality with *in utero* versus *ex utero* transfer ^9, 11, 12^. In a recent UK population study, which compared infants born before 28 weeks’ gestation (n = 17,577), the 2,158 infants transferred within 48 hours of birth to a level 3 unit had no significant difference in the odds of death before discharge (odds ratio [OR] 1.22, 95% CI 0.92–1.61) but significantly higher odds of severe brain injury (OR 2.32, 95% CI 1.78–3.06; NNT 8) and significantly lower odds of survival without severe brain injury (OR 0.60, 95% CI 0.47–0.76; NNT 9) ^9^.

A Canadian national cohort of 28 tertiary neonatal units between 2009 and 2011 reported an excess mortality associated with being transferred *ex utero* (OR 1.33, CI 1.03–1.77) ^12^. Another North American study of *in utero* compared with *ex utero* transfers reported statistically significant increases in the incidence of respiratory distress syndrome, bronchopulmonary dysplasia, intraventricular haemorrhage, and mortality ( *P* = 0.001) ^13^. In a subanalysis of the Swedish population’s level 3 neonatal units, the extremely low birth weight infants who were *ex utero* transfers had a significantly higher mortality than those who were transferred in antenatally (42% versus 26%, adjusted OR 2.8, CI 1.3–5.7) ^11^. However, these benefits are not found across all study populations. In an Australian cohort, there was not a significant difference in mortality within the subset of infants admitted to NICU (29% of *ex utero* transfer infants died within 1 year versus 20% inborn) ^10^. In the EPICURE 2 study, an increased number of babies were transferred within 24 hours of birth, but transfer was not associated with an excess morbidity (unlike in the first EPICURE study cohort). The authors suggest that these improvements may reflect improved, but very costly, neonatal transport services ^14^.

The limitation with many of these retrospective studies is that they include only those who survive to transfer and reach a level 3 NICU, excluding antenatal deaths and deaths before transfer (survival bias). This does not then represent the real impact of an inability to perform *in utero* transfer. An excess of deaths prior to transfer from level 1 or 2 settings is missed by analysing NICU data alone, creating a bias against *in utero* transfer. For example, the EPICURE 2 cohort’s antenatal foetal deaths (32% mortality in level 1 units versus 15% in level 3) contribute significantly to the UK perinatal mortality ^1^. Mothers of extreme preterm infants born in the appropriate setting benefit from tertiary obstetric input and expert delivery room resuscitation. There were significantly more delivery room deaths in level 2 units compared with level 3 units (17% versus 7%, aOR 1.67, CI 1.02–2.72). Lower mortality rates may be associated with greater obstetric expertise at these units, such as appropriate steroid administration and skilled caesarean delivery of the preterm breech. The finding that more women received appropriate antenatal corticosteroid administration in higher-level units supports this assumption ^1^. The contribution of pre-transfer deaths was recognised by Boland *et al.*, who suggested that the perceived poor prognosis at earlier gestations was likely to have prohibited decisions to transfer in referring units (which in fact had a 69% chance of survival if admitted to NICU); in effect, these babies were not given the same chance to survive ^10^.

For the reasons outlined, short of randomising infants to *in utero* or *ex utero* transfer at the point of need for level 3 care is identified (which is both unethical and unfeasible), the true risk of postnatal transfer may be underestimated by some studies. Short of randomized controlled trials (RCTs), propensity score matching to equal the critical background factors in the comparison of outcomes of infants born in different level units is one method of adjusting for these effects, as in the recent UK population study ^9^. Given the substantial evidence regarding mortality and morbidity excess of *ex utero* versus *in utero* transfer and the consensus regarding improved outcomes for those successfully transferred antenatally, compared with those not transferred at all, *in utero* transfer clearly remains the safest policy for women at risk of preterm birth, particularly at early gestation.

## Challenges of *in utero* transfer

### Which mothers need transfer?

**Figure 1.**
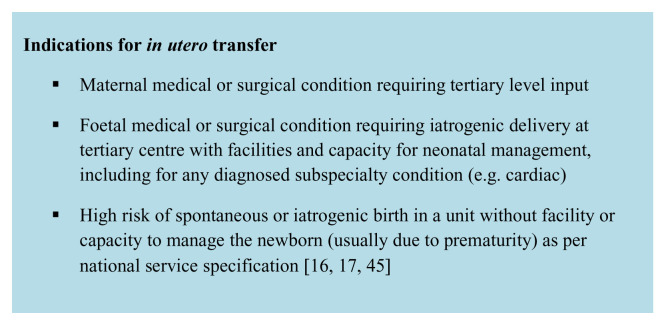
Indications for in utero transfer. This figure was adapted from a previous publication by the authors ^17^ with permission from London Neonatal Operational Delivery Network.

Pregnant women who require *in utero* transfer should be considered to be at high risk of delivery within 7 days for the reasons outlined in Figure 1.

Whilst birth in a NICU centre is clearly optimal at extremely preterm gestations, mothers who are medically unstable (e.g. due to placental abruption or eclampsia) or present in advanced preterm labour are not suitable for ambulance travel owing to the risk of suboptimal clinical monitoring during transfer. Since the maternal risk prevents the transfer of the foetus to an appropriate neonatal facility, such emergency presentations are less likely to receive timely antenatal corticosteroids and are associated with adverse neonatal outcomes. It may be argued, therefore, that these “too unstable to transfer” cases may be responsible for the excess in mortality at services with lower-level neonatal services. Analysis of reasons for transfer render this explanation unlikely because preterm birth and preterm prelabour ruptured membranes are much more common indications than acute maternal conditions ^15^. Furthermore, the high-risk pregnancies most likely to suffer these obstetric complications are over-represented at tertiary (rather than secondary) services because of antenatal referral processes ^16^. In summary, whilst a few of the sickest babies may not be transferred, this is unlikely to wholly account for the substantial excess risk of being born in the “wrong place”.

**Figure 2.**
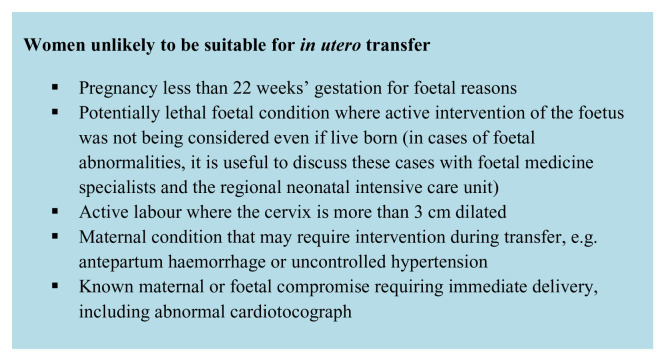
Contra-indications for in utero transfer. This figure was adapted from a previous publication by the authors ^17^ with permission from London Neonatal Operational Delivery Network.

It is, however, critical to reach consensus regarding who is unsuitable for transfer to deliver equitable care to families and to improve transfer processes. Suggested contra-indications collated from UK guidelines are outlined in Figure 2 ^17, 18^. It is recognised that these are context dependent; longer distances or more limited ambulance resources in different settings are likely to impact acceptable levels of risk for *in utero* transfer.

### Inappropriate in utero transfers

Having explored the significance of *in utero* transfers for those who need them, we must also consider inappropriate *in utero* transfers. Between 50 and 80% of *in utero* transfers do not subsequently require neonatal specialist care ^15^. These unnecessary transfers primarily comprise women in threatened preterm labour (TPTL) ^19^. If based on symptoms alone, more than 90% of women transferred for TPTL will not deliver imminently ^20, 21^, so transferring everyone at early gestations in TPTL ^17^ would put additional strain on an imperfect system and endanger successful transfer of those who most need it. In addition, excessive *in utero* transfers could mean that neonatal cots will be harder to find because tertiary units will be unnecessarily “reserving cots” for their own women in false labour. In practice, *in utero* transfers are not performed for all women in TPTL, but aside from National Institute for Health and Care Excellence (NICE) guidelines ^22^, there is little guidance on what an appropriate threshold should be.

A number of predictive tests are available, but only cervicovaginal foetal fibronectin (fFN) measurement and transvaginal ultrasound cervical length measurements demonstrate high predictive performance in robust trials and are recommended by NICE ^22– 24^. Few predictive tests have been clinically evaluated directly in relation to *in utero* transfer. Given the low population and vast distance between hospitals, it is not surprising that Australian research groups have prioritised this issue. As described by Giles *et al.*, in New South Wales, transfers may involve up to a 4-hour air ambulance flight (814 km) and a much higher risk of delivery is felt to warrant transfer (up to 50%) ^25^. This group evaluated the use of qualitative fFN as part of clinical examination in their network over a 2-year period; 98.1% of patients with a closed cervix and a negative fFN did not deliver within 7 days. Importantly, the two women who delivered within 5 days were correctly transferred on the basis of continuing additional symptoms. This study achieved a 51% reduction in transfers. Reduction in transfers saved $153,120 (approximately £85,000), and a reduction in average hospital stay saved an additional $2,970 (approximately £1,640) per patient ^25^.

The QUiPP app is a decision-making tool that provides an individualised percentage risk of delivery (e.g. within 7 days) that is relevant to *in utero* transfer decisions ( https://quipp.org/). QUiPP improves the prediction of preterm birth by combining individual pregnancy characteristics and the continuous variables of quantitative fFN and/or cervical length measurements to better assess risk ^26, 27^. Relative to NICE guidance, it can reduce up to 89% of transfers for women who present prior to 30 weeks’ gestation at hospitals without a NICU ^28^. Its effectiveness at influencing *in utero* transfer decisions is currently under evaluation ^29^.

### What is the burden of in utero transfers on the NHS?

Unfortunately, there are no UK-wide data on the prevalence of *in utero* transfers. An audit of activity in Greater Manchester reported an incidence of 8.5 *in utero* transfers per 1,000 deliveries ^19^. In London, when urgent neonatal transfer requests increased between 2001 and 2004, there was a significant decrease in antenatal transfer requests (relative risk [RR] 0.60, *P* <0.01) and an increase in *ex utero* transfers, which has been attributed to the introduction of a centralised neonatal transport service in 2003, creating complacency regarding the urgency of *in utero* transfers ^30^. Data provided from the Emergency Bed Service (who provide assistance with cot location in London) suggest that *in utero* transfer requests declined from over 100/month in 2004 to 68/month in 2012 ^31^.

NHS Scotland conducted a national review of all of its *in utero* transfers over a 6-month period. There were a total of 599 *in utero* transfers, 72% of which were from community maternity units to consultant-led units and 28% (n = 165) were from consultant-led units to consultant-led units, half of which (n = 86) were between tertiary units ^32^. The high contribution of *in utero* transfers for capacity rather than expertise is widespread in the UK. Such transfers may be associated with less excess morbidity and mortality but still entail significant stress and cost for the parents. Transfers for expertise are inevitable with neonatal subspecialisation, but transfers due to capacity alone are indicative of a dysfunctional over-stressed system.

By definition, *in utero* transfer is difficult to evaluate and organise owing to reporting systems and clinical pathways that do not cross providers or specialties (obstetrics, midwifery, and neonatology) adequately. The lack of ownership around this challenging process has no doubt contributed to the lack of progress in improving the system in recent decades. However, recent NICE preterm birth guidance advises that all women in TPTL who are <30 weeks’ gestation should be transferred to units with appropriate neonatal expertise (without the use of predictive tests) ^16, 22^. If widely adopted, this guidance will dramatically increase the total number of *in utero* transfers, without prudent prioritisation of the most important ones.

Transfers are time-consuming and costly because of inefficient transfer processes. In the London Neonatal Transport Teams’ detailed analysis of *in utero* transfers, 47% (158/338) of referrals to the emergency bed service were not successful, and 11/69 postnatal transfers prior to 29 weeks’ gestation were identified as failed *in utero* transfers. Clinicians spent a median (interquartile range) of 240 (150–308) minutes contacting seven (6–8) units when trying to arrange transfers ^33^. The high occupancy levels of neonatal units and the shifting capacity of maternity units are cited as the primary reasons for difficulty in locating a suitable unit to refer to ^24, 25^. A lack of utilisation of technology or a unified referral process compound this problem according to a UK survey of clinicians involved in this process ^31^. In the North West region, where a dedicated cot bureau exists, the three most common reasons for failed *in utero* transfers were a lack of maternal beds (29%), a lack of cots (23%), and transfer deemed inappropriate following consultant-to-consultant discussion (26%) ^19^.

The administrative burden and pathway variation of *in utero* transfer risk compromising the quality of clinical care. Antenatal corticosteroid administration is likely to have optimal impact on reducing neonatal mortality and respiratory distress syndrome if given between 24 hours and 7 days of delivery ^34, 35^. In the context of *in utero* transfer, it is common for the first dose to be delayed until the mother reaches the receiving unit and for steroid courses to be incomplete because of communication failures ^19^.

### Impact of *in utero* transfers on women

It is well recognised that the transfer of babies in the early neonatal period is a frightening and stressful experience for parents ^35^. The loss of familiarity and separation from their vulnerable child at a critical stage when bonds are being established represent a disruption in their parenthood, which can impair the health and development of the infant ^37^. It is important to emphasise that for parents whose babies are delivered immediately after transfer, the *in utero* experience avoids separation from the baby and is preferable to a postnatal transfer. However, transfers before birth are also associated with emotional and financial burden upon the women and their families because of longer travel distances between home and their newborn. Broadly, women recognise the importance of *in utero* transfer and find it acceptable ^15, 38^. However, the unplanned relocation to unfamiliar surroundings at an already stressful time in the pregnancy creates anxiety, shock, and worry ^38^. The emotional and financial burden of *in utero* transfers upon women and their families is beginning to be explored in the literature, such as the following description of a woman who was transferred from her home hospital because of capacity issues:

“ *A nightmare. Not something I'd like to relive. Because although there wasn't massive complications or anything, I got really stressed because I didn't know what was going to happen… And I think it's quite annoying, because I think me and my partner spent quite a lot of time getting annoyed, thinking why couldn't he have just stayed at XXX*” ^38^.

Pregnant women value choice of birth place and continuity of care as important aspects of their care ^39^, and *in utero* transfer can feel like the antithesis of this. The loss of choice and control associated with relocation may be driven by local cultural identities as much as inconvenience ^40^.

There is also considerable anxiety around domestic and logistical issues. Separation from children left at home is one of the most difficult aspects reported, and many perceived a particularly negative and unrecognised impact on their partners ^38^. The travel costs, excess phone bills, accommodation, and unpaid leave for partners add up to considerable financial strain. The excess strain of being transferred elsewhere compared to a local admission for TPTL would benefit from a direct comparison of women’s experiences, as some of the domestic and financial issues would be incurred with any admission during pregnancy.

Given that stress is a known predictor of adverse pregnancy outcomes ^41, 42^, the excess anxiety caused by transfer may have a negative impact on both mother and baby. Some of the qualitative studies allude to methods by which we could improve the *in utero* transfer process for women, such as improved communication or financial remittance to transferred women ^38^. Therefore, improving TPTL risk assessment and *in utero* transfer pathways may help in shared decision-making regarding the need for *in utero* transfer and have a significant impact in reducing the anxiety of the whole experience. Critically, for families whose infants do not go on to deliver imminently, better selection of the most appropriate women for transfer would avoid the parental strain of *in utero* transfer altogether for some families.

## Reforming the *in utero* transfer process

The negative impact on women’s pregnancy experiences, inappropriate selection of the right women to transfer, and high administrative costs all demand time for change.

### Learning from others: centralised transfer services

Following their comprehensive audits of the problem, cot bureaus have been established in some regions of the UK ^19, 32, 43^. As well as regularly phoning neonatal units to check cot status, the cot bureaus liaise with neonatal and maternity teams to arrange referrals for particular requests. Centralising the referrals reduces the clinical time spent on organising transfer, establishes a unified process across hospitals, and is likely to create efficiencies due to developing expertise in the problem. However, even within these dedicated services, around one-third of transfer requests are unsuccessful because of capacity issues, withdrawal of requests, or a lack of consensus on the appropriateness of the *in utero* transfer ^20, 33, 43^. This suggests culture change amongst referrers and receivers of *in utero* transfers is needed in addition to further investment in capacity.

Centralisation of perinatal services to five tertiary centres in Finland from 1999 to 2017 has been associated with increases in survival of very preterm infants (<32 weeks’ gestation) from 72% in 1987 to over 90% since 2012. This has been achieved with a very low rate of *ex utero* transfers (2–4% of all very preterm infants) ^8^. How this was achieved may be linked with the Finnish national birth register, which empowers clinicians to implement evidence-based change from the high-quality data they themselves have generated ^8^. The smaller population, and a well-funded government health system, may also facilitate centralising expertise and strike a contrast with the UK.

This differs to Australia, where variations in resources and geography are more profound. Policy makers have decided to prioritise the issue of *in utero* transfer in this setting. State- and territory-wide policies vary slightly according to local resources and distance to referring hospitals but differ little on risk stratification and management. The common theme amongst the more streamlined services is a centralised coordinated approach. Victoria utilise the Paediatric Infant Perinatal Emergency Retrieval (PIPER) service. They offer a one-point-of-contact 24-hour emergency conference line involving a dedicated PIPER neonatal consultant and retrieval team. The team provide advice where required, organise an appropriate referral site, and mobilise a team for transfer. Queensland transfers are facilitated by Retrieval Services Queensland (RSQ), who provide a 24-hour call service dedicated to identifying suitable accepting units and initiate a conference call between referring and accepting obstetric teams ^44^.

Rural Western Australia has only one tertiary referral hospital serving an area of over 2.5 million square kilometres, so aeromedical retrieval is the preference for the vast majority of *in utero* transfers. Interestingly, there has never been a preterm delivery mid-flight despite women regularly being transferred in established preterm labour. The possible effect of ambient altitude and cabin pressure on delaying preterm delivery has been hypothesised as a possible explanation for this ^33^.

### Changing policy in the UK

**Figure 3.**
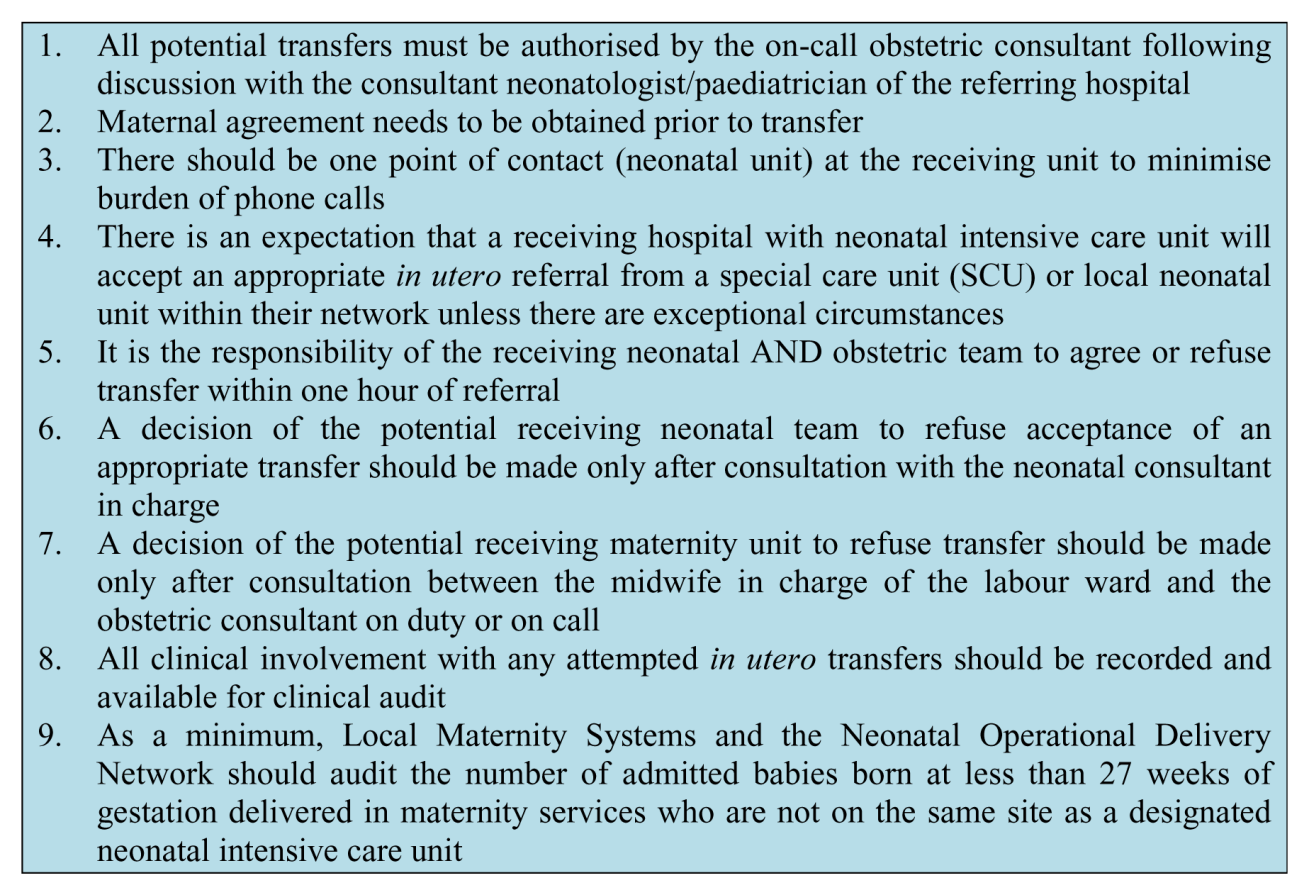
Main messages and recommendations reported in the Pan-London In Utero Transfer Guidance: Changing the Conversation. This figure was adapted from a previous publication by the authors ^17^ with permission from London Neonatal Operational Delivery Network.

Following extensive stakeholder collaboration, an NHS England pan-London working party on *in utero* transfer concluded that improved, unified data collection across the region was a key target and an *in utero* transfer record was created for this purpose (see Figure 3) ^17^. The goal of this regional guideline is to better inform clinicians, support their decision-making process, and reduce inappropriate variation in practice. A core theme across the recommendations was to change the culture around the perception of *in utero* transfers being “not my problem” and encouraging an opt-out rather than an opt-in approach. For example, a lack of a readily available bed on the labour ward ought not always to preclude transfer, as co-ordinators are adept at directing the flow of women through maternity services and many women could transfer to an antenatal ward initially.

The objectives and recommendations of this London-based guideline might provide a useful template for other UK regions, although variations in networks, resources, and geography would require that the creation of a national *in utero* transfer guideline had extensive stakeholder involvement, including patients and their families. For example, NHS Scotland’s *in utero* transfer review prompted staffing reviews across all level 3 units and recommendations regarding the use of tocolytic therapy and the use of predictive tests to accurately diagnose preterm labour. This was aligned with their centralised system to identify available beds ^32^.

### Technological solutions

Whilst improving transfer protocols and inter-hospital pathways is fundamental to addressing problems with *in utero* transfer, a 2013 survey of UK clinicians found that a lack of utilisation of technology was viewed as a significant contributor to low success rates ^31^. Technological solutions can offer a real-time transparent overview of cot availability facilitating centralised coordination of neonatal services. In Australia, New South Wales Health have pledged $1.5 million (£842,000) to enhance current transfer processes. In addition to provision of a 24/7 perinatal advice line, the Maternal Transfers Redesign Project plans to implement an Electronic Patient Journey Board with maternity and neonatal functionality. The system will provide a real-time dashboard allowing for allocation and monitoring of maternity and neonatal beds across the state.

The Scottish “Scotstar” neonatal transport system co-ordinates *in utero* transfer requests alongside *ex utero* perinatal transfer requests across Scottish neonatal networks ( https://www.neonataltransport.scot.nhs.uk). Having taken the clinical details from the referrer, the cot bureau uses BadgerNet cot locator to identify likely cot availability before contacting units. Scotland’s uptake of the BadgerNet maternity platform as well as the neonatal platform enhances continuity between obstetric and neonatal events, such as transfer, relative to less compatible digital systems. As the BadgerNet software is now utilised for computerised neonatal records in 250 hospitals across the UK, Australia, and New Zealand, there are significant synergies from using an already acceptable and integrated platform.

**Figure 4.**
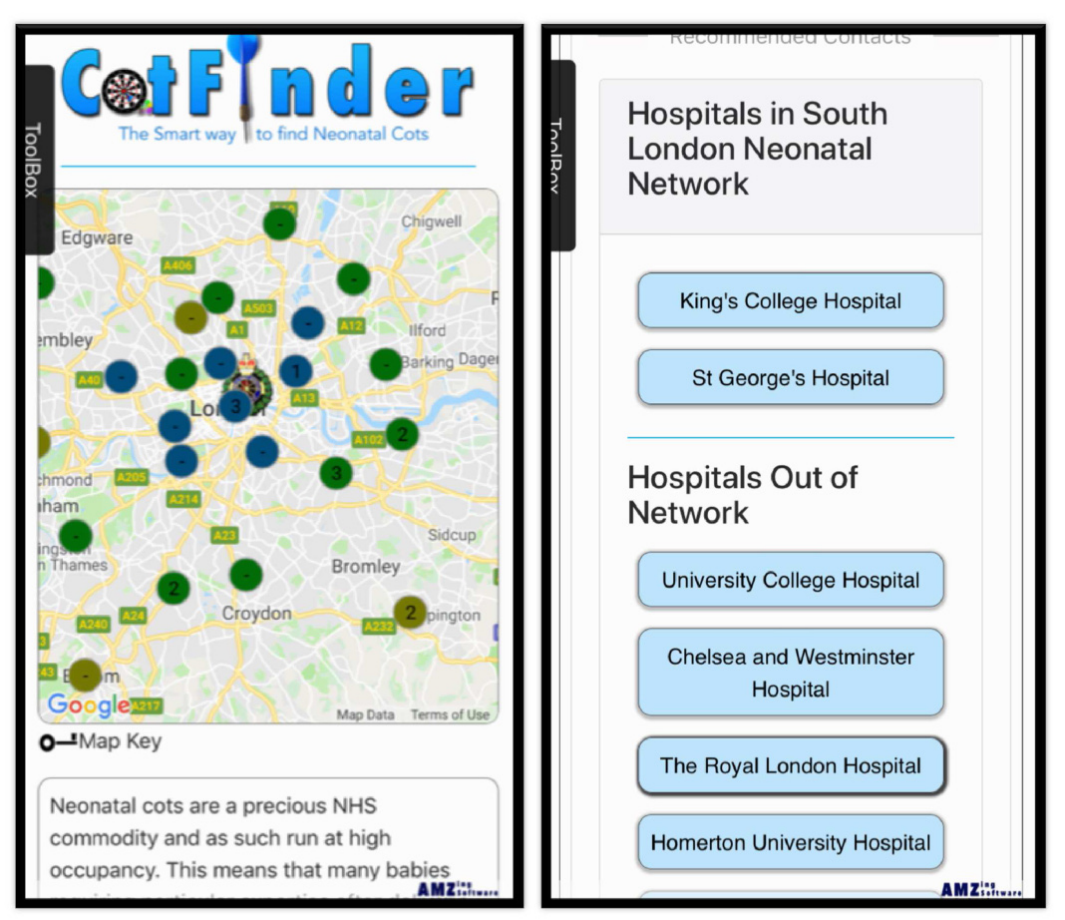
CotFinder app screenshots.

With the advent of smartphones, there is the potential to deliver *in utero* transfer reform from the palm of the clinician’s hand. Conceived and created by UK obstetric and gynaecology junior doctors, a free android app, iOS app, and website is now available to streamline the *in utero* transfer process using GPS technology and neonatal network algorithms to provide a live database of cot availability in the surrounding hospitals ( https://www.cotfinder.com/) ( Figure 4). Search results are arranged in order of relevance (network, expertise), and the relevant contact details of both the neonatal unit and the maternity unit are provided. It also has the capability to record requests, tests, and outcomes to ease future audit of this problem.

If commitments to update the cot status can be secured, CotFinder has the potential to transform the ease and success of *in utero* transfer throughout the UK and beyond.

CotFinder’s appropriateness has been confirmed by 100% daily updates in South London Neonatal Operational Delivery Network following roll-out in August 2019. However, its successful wider adoption depends on a cultural shift towards greater shared responsibility and investment in this issue.

## Conclusion

*In utero* transfer is an unavoidable aspect of maternity and neonatal care, the burden of which is likely to increase if preterm birth rates rise, attitudes change towards viability at thresholds of survival, and health services continue to face funding pressures. Competing inter-disciplinary tensions between valid concerns for neonatal wellbeing and the risks and burden of both necessary and unnecessary transfer have led to inconsistent practices. Policy reform of inter-hospital transfers needs to be regional, if not national, by definition. Emerging change in attitudes to this shared responsibility coupled with technological innovation offer real promise for transformation. By better prioritisation of *in utero* transfers, obstetric teams have an opportunity to demonstrate how far their advocacy for pregnant women and their babies goes beyond the delivery room. Sustained multi-disciplinary commitment with buy in from obstetric, midwifery, and neonatal teams is crucial to effective planning for preterm delivery to maximise good outcomes.
